# Self-assembling peptides with hBMP7 biological activity promote the differentiation of ADSCs into nucleus pulposus-like cells

**DOI:** 10.1186/s13018-022-03102-8

**Published:** 2022-04-02

**Authors:** Chaofeng Wang, Zhong Li, Kun Zhang, Congming Zhang

**Affiliations:** Department of Orthopaedic Surgery, Hong Hui Hospital, Xi’an Jiaotong University College of Medicine, Xi’an, China

**Keywords:** Self-assembling peptide, BMP-7 short peptide, Nucleus pulposus cell, Tissue engineering

## Abstract

Functionalized self-assembling peptides, which display functional growth-factor bioactivity, can be designed by connecting the C-terminus of a pure self-assembling peptide with a short functional motif. In this study, we designed a novel functionalized peptide (RADA16-SNVI) in which an SNVI motif with hBMP-7 activity was conjugated onto the C-terminus of the RADA16 peptide via solid-phase synthesis. A mix of RADA16-SNVI and RADA16 solutions was used to create a functionalized peptide nanofiber scaffold (SNVI-RADA16). The hydrogels were analyzed by atomic force microscopy, circular dichroism, and scanning electron microscopy. The results showed that the SNVI-RADA16 solution effectively formed hydrogel. Next, we seeded the SNVI-RADA16 scaffold with adipose-derived stem cells (ADSCs) and investigated whether it displayed biological properties of nucleus pulposus tissue. SNVI-RADA16 displayed good biocompatibility with the ADSCs and induced their expression. Cells in SNVI-RADA16 gel had a greater secretion of the extracellular matrix marker collagen type II and aggrecan compared to ADSCs grown in monolayer and control gel (*p* < 0.05). The ratio of the aggrecan to collagen in cells in SNVI-RADA16 gel is approximately 29:1 after culture for 21 days. ADSCs in SNVI-RADA16 gels expressed the hypoxia-inducible factor 1α(HIF-1α) mRNA by real-time PCR. However, HIF-1 mRNA is absence in control gel and monolayer. The results suggested that the functionalized self-assembled peptide promotes the differentiation of ADSCs into nucleus pulposus-like cells. Thus, the designed SNVI-RADA16 self-assembling peptide hydrogel scaffolds may be suitable for application in nucleus pulposus tissue regeneration.

## Introduction

Disc degeneration disease (DDD) is a musculoskeletal spinal disorder resulting in substantial pain and dysfunction and a major cause of morbidity [1, 2]. The main treatments for DDD are conservative therapy and surgical therapy. Surgeries include discectomy and spinal fusion. However, surgical treatment is associated with complications such as altering the natural and physiologic functions of the intervertebral discs (IVD) and accelerating the adjacent IVD degeneration [3, 4].

Recently, tremendous progress has been made in stimulating the regeneration of the intervertebral disk by using biological approaches including tissue engineering [5–7]. Nucleus pulposus (NP) tissue engineering may offer a solution to disc degeneration. However, to date, traditional in vitro tissue engineering techniques have not been able to satisfactorily establish regeneration of the IVD from the NP tissue because the discs show further degeneration when the annulus fibrosus is incised for transplantation of the NP tissue. Cell-based therapies, such as NP cell injection, are conceived as more ideal therapeutic strategies for degenerated disc repair.

The synthetic peptide RADA16 (AcN-RADARADARADARADA-CONH2) is one of the most commonly used self-assembling nanofiber peptides [8], and it has been successfully used to create biomaterials for tissue engineering and regenerative medicine. RADA16 peptides exhibit good biocompatibility and bioactivity [9–11] and can quickly assemble into nanofibers that form hydrogels with over 99% water content and 5–200 nm pore sizes, which are structurally similar to natural extracellular matrix (ECM). As RADA16 is composed of synthetic amino acids, it can be tailored to specific functional requirements [12]. A variety of bioactive short peptide motifs that act as growth factors or ECM macromolecules have been conjugated to the C-terminus of RADA16 to promote cell migration, proliferation, and differentiation [13, 14].

Transforming growth factor-β (TGF- β) [15, 16] was shown to have the potential to enhance cartilage formation. Bone morphogenetic protein-7 (BMP-7), an important member of the TGF- β superfamily, has been found to not only stimulate the synthesis of proteoglycans and collagen type II in vitro [5, 17], but also increase the expression of ECM genes and the disc height of degenerated disc in rabbits [18]. Takashi [19] and Alper [20] reported respectively that TGF-β1 and BMP-7 could co-regulate the ECM secretion. In our study, we assume that TGF-β1 and BMP-7 could co-regulate ADSCs differentiation into nucleus pulposus-like cells.

More recently, Chen and Webster [21] found that the short functional peptide SNVI (SNVILKKYRN), which originates from the bioactive sequence of BMP-7, displayed BMP-7 bioactivity. More importantly, SNVI could easily be chemically functionalized onto nanoscale biomaterials.

To avoid quick degradation of the growth factors resulting in loss of bioactivity, in this study, we designed a novel functionalized peptide (RADA16-SNVI) in which the SNVI motif with human BMP-7 (hBMP-7) biological activity was conjugated to the C-terminus of the RADA16 peptide via solid-phase synthesis. A mixture of functionalized RADA-SNVI and RADA16 solutions was used to create a functionalized peptide nanofiber scaffold. In addition, we constructed injectable ADSC-seeded functionalized scaffold and investigated whether it showed NP biological properties. Finally, we investigated whether the functionalized self-assembling peptides facilitated the differentiation of ADSCs into NP-like cells.

## Materials and methods

### Synthesis of self-assembling peptides

**Fig. 1:**
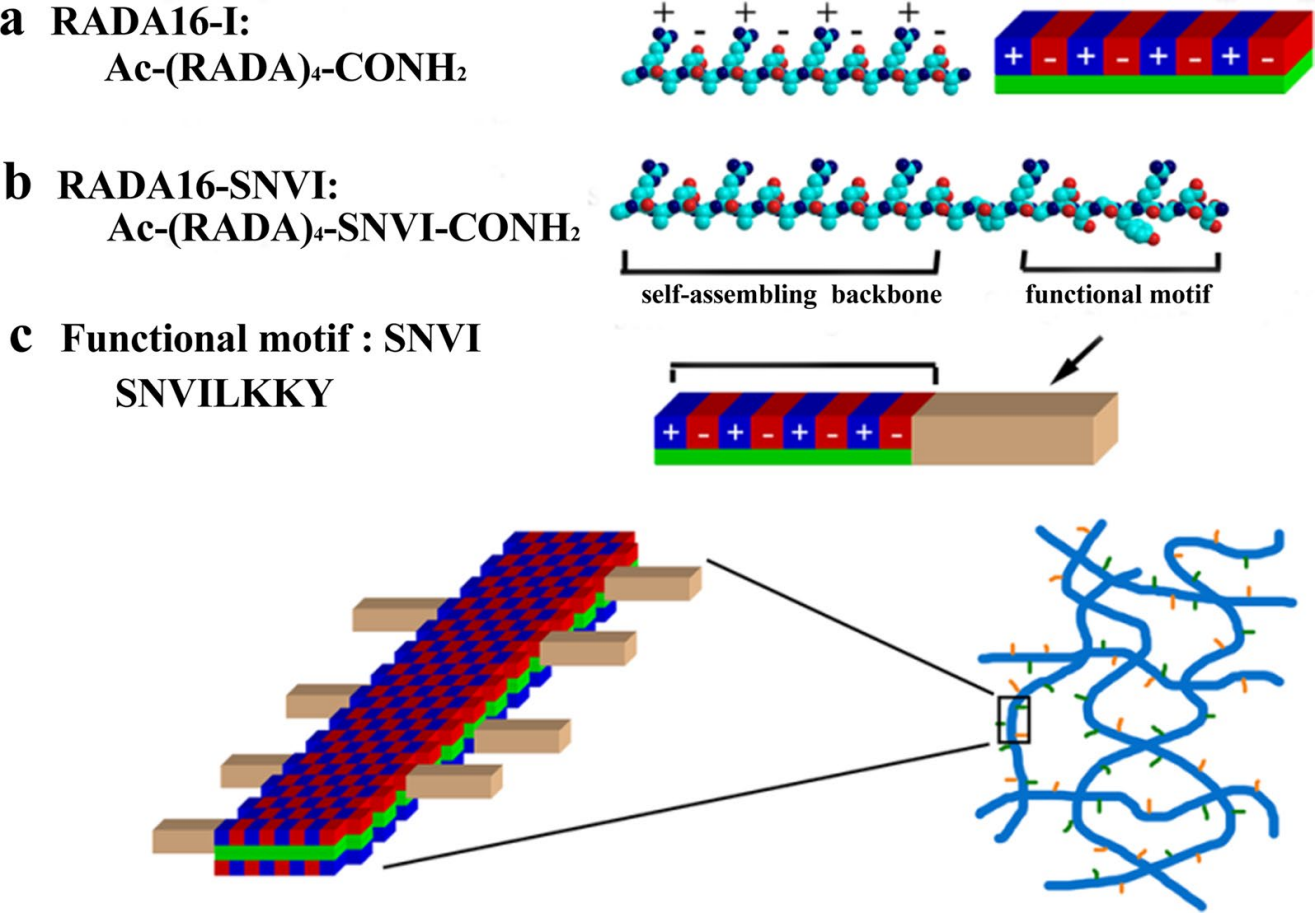
Molecular models of the self-assembling peptide RADA16 and the modified self-assembling peptides RADA16-SNVI. **a** Amino acid sequence, molecular model, and secondary structure of RADA16. **b** Amino acid sequence, molecular model, and secondary structure of RADA16-SNVI. **c** Amino acid sequence of the functional motif SNVI

We designed a functionalized self-assembly peptide RADA16-SNVI (AcN–RADARADARADARADA-GG-SNVILKKYRN-COHN2) by conjugating the N terminus of SNVI (SNVILKKYRN) to the C-terminus of the self-assembling peptide RADA16 (AcN–RADARADARADARADA–COHN2) with a two-glycine (G) residue spacer to ensure stable self-assembly without disturbing the biological function of the SNVI fragment (Fig. 1). The RADA16 (purity ≥ 98%) and RADA16-SNVI peptides (purity ≥ 95%) were synthesized and purified by the commercially company (Sangon Biotech Co., Ltd. China). The purity of the peptides was confirmed by high-performance liquid chromatography (HPLC). For hydrogel creation, the peptide powders were dissolved in a sterile 10% sucrose solution at a final concentration of 1% (w/v) and sonicated for 30 min. The final concentration of 1% (w/v) SNVI-RADA16 mixture was mixed with 2% RADA16-SNVI solution and 2% RADA16 solution at a ratio of 1:1. Two hundred microliters of the mixtures were transferred to Transwell microplate membrane inserts (Millipore, USA) that were carefully added to the wells of 24-well plates containing 600 μl culture medium for gelation at 37 °C for 30 min. The medium was then changed for further incubation for 30 min to enhance self-assembly and to equilibrate the hydrogel to physiological pH.

### Structural analysis

In accordance with Tao’s study, atomic force microscopy (AFM) was used to analyze the nanofiber structure. All of the peptide solutions were diluted to 0.01% (w/v). Five microliters of each diluted sample was dropped onto a freshly cleaved mica surface and left for 5 s, and then the surface was gently rinsed twice using 100 μL of distilled water. The peptide samples on the mica surface were then air-dried at room temperature for 3 h. Images of the samples were acquired using an AFM (SPA-300 HV, Seiko Instruments, Japan). The scanning area was 1 × 1 μm, and the frequency was 1.02 Hz.

The secondary structure of peptides was studied using circular dichroism (CD). The 50 μM peptide sample by diluted to a 0.1%(w/v) was incubated at room temperature overnight. The CD spectra were collected using a quartz curet with a path length of 0.5 cm, in a wavelength range of 195–250 nm. The scanning speed is 100 nm/min and spectral band width is 1.0 nm. Each spectrum was collected in five times.

The microstructure of the self-assembly peptide was observed by scanning electron microscopy (SEM). All of the self-assembling peptide hydrogel scaffolds were fixed, dehydrated, and coated with platinum. The images were captured using a JEOL SEM at 800–24,000 × magnification at a voltage of 20 kV.

The rheological behavior of the peptide samples was evaluated using a RheoStress AR G2 instrument (TA Instruments, Inc., USA). The mixture of 1% peptide solution and DMEM at a ratio 2:1 was immediately loaded on the lower plate. During this process, the storage (elastic) modulus (*G*′) and loss (viscous) modulus (*G*′′) of the designer self-assembling peptides were evaluated in the frequency sweeps ranging from 0.1 to 100 rad/s, which were performed at a constant shear stress of 1 Pa at 37 °C.

### ADSC cell culture and hydrogel seeding

The animal experiment protocol was approved by the animal ethics committee of Navy General Hospital of China. Adipose tissue was harvested from the inguinal region of a 1-year old beagle dog after sterilization of the skin surface. The adipose tissue-derived stem cells (ADSCs) were split routinely when nearing confluence. The ADSCs were suspended in 20% sucrose at a concentration of 5 × 10^5^ cells/ml before seeding. For three-dimensional cell culture, 100 μl of ADSC suspension was mixed with 100 μL of the different peptide solutions. The mixtures (200 μl) were immediately transferred to Transwell inserts (Millicell-CM, 10-mm diameter, Millipore, USA). Transwell inserts containing the mixtures of ADSC-peptide were placed into the wells of a 24-well culture plate. Then, 500 μl of culture medium was added to per well. Transwell inserts were covered completely by culture medium to avoid exposing to air and incubated at 37 °C for 30 min. The medium was changed for further incubation for 30 min to enhance self-assembly and to equilibrate the hydrogel to physiological pH. After 1 h of incubation, 500 μl of DMEM/F-12 medium containing 10% fetal calf serum (Gibco, USA), 10 ng/ml TGF-β1 (Gibco, USA), streptomycin (Gibco, USA), and penicillin G (Gibco, USA) was added to each well. The medium was replaced every 3 days. The hydrogels were harvested after culture for 7, 14, and 21 days. 100 ml ADSCs suspension at a concentration of 5 × 10^5^ cells/ml were added to 24 well plates as control groups. And 500 ml culture medium was added to every well. The medium was replaced every 3 days. The cells were harvested after culture for 7, 14, and 21 days. Monolayer cultures of ADSCs grown under the same culture conditions were used as controls.

### Cell attachment and viability in the functionalized hydrogel scaffolds

To observe the attachment and viability of the ADSCs encapsulated in the peptide hydrogel scaffolds, the ADSCs were mixed with the pure RADA16 and SNVI-RADA16 mixture solutions to form hydrogels. By days 14, live viability assay was performed using a Live/Dead assay (Molecular Probes, Life Technologies, CA, USA). The cell/hydrogel sample was treated with serum-free medium containing 2 μM calcein AM and 4 μM ethidium-1 at 37°℃ for 15–20 min. After removal of staining solutions and washing with PBS, cells were observed under confocal laser scanning microscopy and analyzed by “Image J” software. SEM was used to observe the attachment of ADSCs to the scaffolds at 14 days. The hydrogels were fixed for 12 h at 4 °C in a 2.5% glutaraldehyde fixation fluid, dehydrated, and coated with platinum. The images were obtained with a JEOL SEM at an accelerating voltage of 20 kV and a magnification of 800–24,000 × .

### Real-time PCR

To assess the effects of the different self-assembly peptides on the ADSCs, the mRNA levels of collagen type I and II, and aggrecan in the three groups (RADA16, SNVI-RADA16 and monoculture) were assayed by real-time PCR at 7, 14, and 21 days. The threshold cycles (Ct) of collagen type I and II, and aggrecan were standardized to that of GAPDH. The amount of mRNA in the hydrogels at 7, 14, and 21 days was compared to that in the control ADSC samples. The primers used in this study are listed in Table 1. The results were expressed relative to control ADSCs that were cultured in culture plates.

**Table 1:** Primer sequences for collagen Type I, collagen Type II, aggrecan and GAPDH

Target gene	Forward primer (5′–3′)	Reverse primer (3′–5′)
Collagen type I	5′ AAGAAGAAGACATCCCACCAGTC 3′	3′ ACAACACGCTACTGCACTAGA 5'
Collagen type II	5′ CCCGAACCCACAAACAACA 3′	3′ CCGAGACGTGACTTACCGA 5'
Aggrecan	5′ CCTACGATGTCTACTGCTATGTGG 3′	3′ CTTAGAGTATTGCGGTGGGAC 5'
GAPDH	5′ ATGTTTGTGATGGGCGTGAA 3′	3′TGACGAACCGAGGAGATCGG 5'

### Detection of hypoxia-inducible factor-1α (HIF-1α) gene expression by RT-PCR

Different Hydrogels contained cells and ADSCs were collected respectively after 21 days culture. Total cellular RNA was isolated using Trizol reagent (Hyclone, Logan, Utah, USA). Total RNA was extracted using the RNA Easy kit (Tiangen Biotech Co. Ltd., Beijing, China) according to the manufacturer’s instructions. PCR was performed with Taq DNA Polymerase (Takara, Tokyo, Japan) using a pair of primers for canine HIF-1αwith an expected product length of 582 bp, (forward primer: HIF-1α-F 5′-TCTGAGGGGACGCGAGGAT-3′ reverse primer: HIF-1α-R: 3′-CCTGGTCCACAGAAGATGTTT-5′). Canine glyceraldehyde-3-phosphate dehydrogenase (dGAPDH) was amplified as an internal control for RNA loading (Gen- Bank accession number: L-23961; forward primer: GAPDH-F: 5′ GATGCTGGTGCTGAGTATGT 3′, reverse primer: GAPDH-R: 3′ GAAGCCGTAGCACCTC 5′, expected product length: 252 bp). PCR products were separated in 1.5% agarose gel.

### Enzyme-linked immunosorbent assay (ELISA) of collagen type I and II, and aggrecan

ADSC-seeded hydrogels and control ADSCs were cultured for 7, 14, and 21 days. Then, the hydrogels and ADSCs were homogenized in a lysis buffer supplemented with a protease inhibitor cocktail (Roche, USA). The supernatants of the different samples were transferred to Eppendorf tubes. After heat denaturation at 95 °C for 5 min, the supernatants were used to assess the amount of target protein by Canine Col I, Col II, and Aggrecan ELISA kits (RD). Standard curves were constructed using serial dilutions of canine collagen I and II, and aggrecan (RD, USA) dissolved in 0.05 M carbonate/bicarbonate buffer. One hundred microliters of the supernatants and standard samples were added to each well of a 96-well plate, in triplicate. The amount of collagen type I and II, and aggrecan in each sample was quantified using the standard curve, according to the manufacturer’s instructions.

### Statistical analysis

All ADSCs were harvested from the same dog donor. All the experiments were repeated to be performed five times. All the data are expressed as the mean ± standard deviation (SD). Homoscedasticity of the data was determined using a homogeneity of variance test. In case of homoscedasticity, two-way analysis of variance (two-way ANOVA) was used to find differences between the groups and times, followed the post-hoc multiple comparisons by Fisher’s least significant difference test to compare the means of each group. In case of unequal variances, Tamhane’s T2 was used for multiple comparisons. *p* < 0.05 was considered significant.

## Results

### Self-assembly of the peptides

**Fig. 2:**
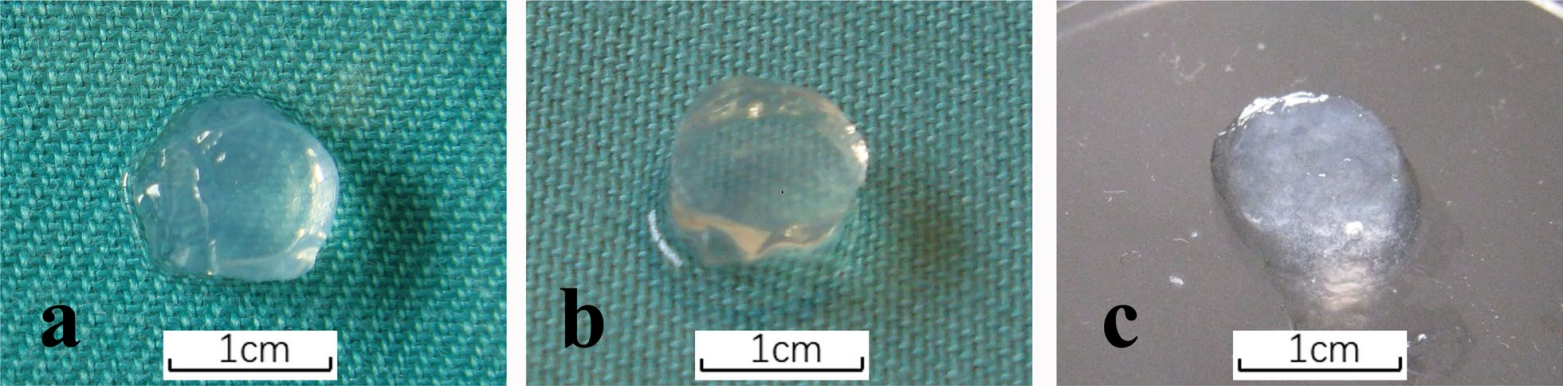
Hydrogel formation from different peptide solutions. **a** Hydrogel formation from a mixture RADA16 and RADA16-SNVI solutions. **b** Hydrogel formation from RADA16. **c** Inefficient hydrogel formation from RADA16-SNVI solution

In order to develop novel self-assembling peptide scaffolds with hBMP-7 biological activity, the original RADA16 was directly extended with SNVI at the C-terminus through solid-phase synthesis. The RADA16 and SNVI-RADA16 mixture peptide solutions formed transparent and elastic hydrogels, but the RADA16V-SNVI solution did not allow effective hydrogel formation (Fig. 2). Therefore, we selected the RADA16 and SNVI-RADA16 mixture solutions for further study.

### Structural analysis of the self-assembling peptides

**Fig. 3:**
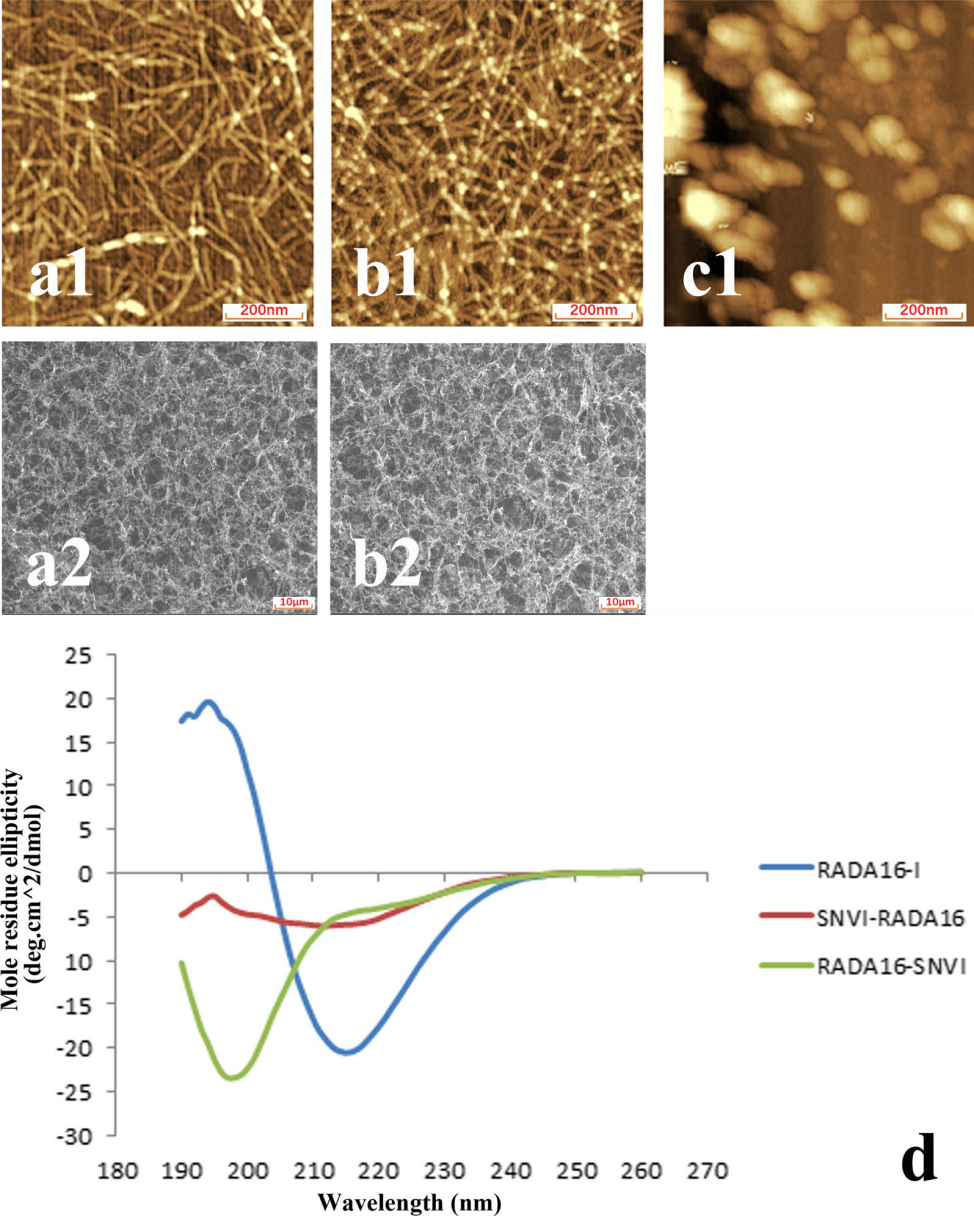
AFM images, SEM images, and CD spectra of the three peptide solutions. AFM (**a1**) and SEM (**a2**) images of the SNVI-RADA16 solution. AFM (**b1**) and SEM (**b2**) images of the RADA16 solution. **c1** AFM image of the RADA16-SNVI solution. **d** CD spectra of the three solutions: RADA16 and SNVI-RADA16 show a typical β-sheet spectrum, while RADA16-SNVI does not

The microstructural characterization of the peptides was carried out using AFM, CD, and SEM. The AFM images showed uniform and interweaved nanofibers both in the RADA16 and the SNVI-RADA16 mixture, but not in RADA16-SNVI solution (Fig. 3a). The diameter and length of the nanofibers in these self-assembling peptides differed. The RADA16 solution formed nanofibers at a higher density and with a smaller diameter (14.2 ± 1.3 nm) than the SNVI-RADA16 mixture (30.5 ± 2.2 nm). The length of the nanofibers ranged from several hundreds of nanometers to a few micrometers. From the AFM result, SNVI functional motif hinders the formation of nanofibers. The RADA16/SNVI-RADA16 mixture has formed the nanofibers similar to RADA16 alone. But the nanofibers of the RADA16/SNVI-RADA16 mixture are lower density and the bigger diameter than that of the RADA16.

The RADA16 peptides displayed a typical β-sheet CD spectrum with a negative maximum molar residue ellipticity (deg cm^2^/dmole) at 216 nm and a positive maximum at 195 nm (Fig. 3d). The SNVI-RADA16 mixture peptide solution similarly displayed a β-sheet spectrum. However, compared to the RADA16 solutions, the intensity of molar residue ellipticity at 216 nm and at 195 nm was decreased in the SNVI-RADA16 mixture solutions and the β-sheet content was reduced. This might have been caused by random coil of the RADA16-SNVI functional peptide. The RADA16-SNVI peptide solution showed no β-sheet structure, but exhibited a spectrum typical for random-coil structures.

The SEM analysis confirmed the nanofiber formation of self-assembled peptides in the RADA16 and SNVI-RADA16 mixture solutions (Fig. 3a, b). The results showed that the RADA16 nanofibers were interwoven into a porous structure with 15 nm diameter and 5–200 nm pore size, while the SNVI-RADA16 nanofibers formed a porous structure with 30 nm diameter and 20–250 nm pore size. Because the porous structures were similar to that of natural ECM, we hypothesized that they would allow tight attachment of the cells to the scaffold and optimal nutrition exchange.

The frequency sweep results revealed that the viscoelasticity of RADA16 and SNVI-RADA16 Hydrogel was similar, and both *G*′ and *G*′′ were independent of the shear rate frequency. A typical gel-like behavior was evident as *G*′ values (100 Pa) of RADA16 and SNVI-RADA16 hydrogels were approximately one order of magnitude greater than *G*′′ values (10 Pa) in the range of the shear rate frequencies. In addition, the values of *G*′ and *G*′′ in RADA16 were greater than the SNVI-RADA16 peptides.

### Attachment and viability of the ADSCs in the functionalized hydrogel scaffolds

**Fig. 4:**
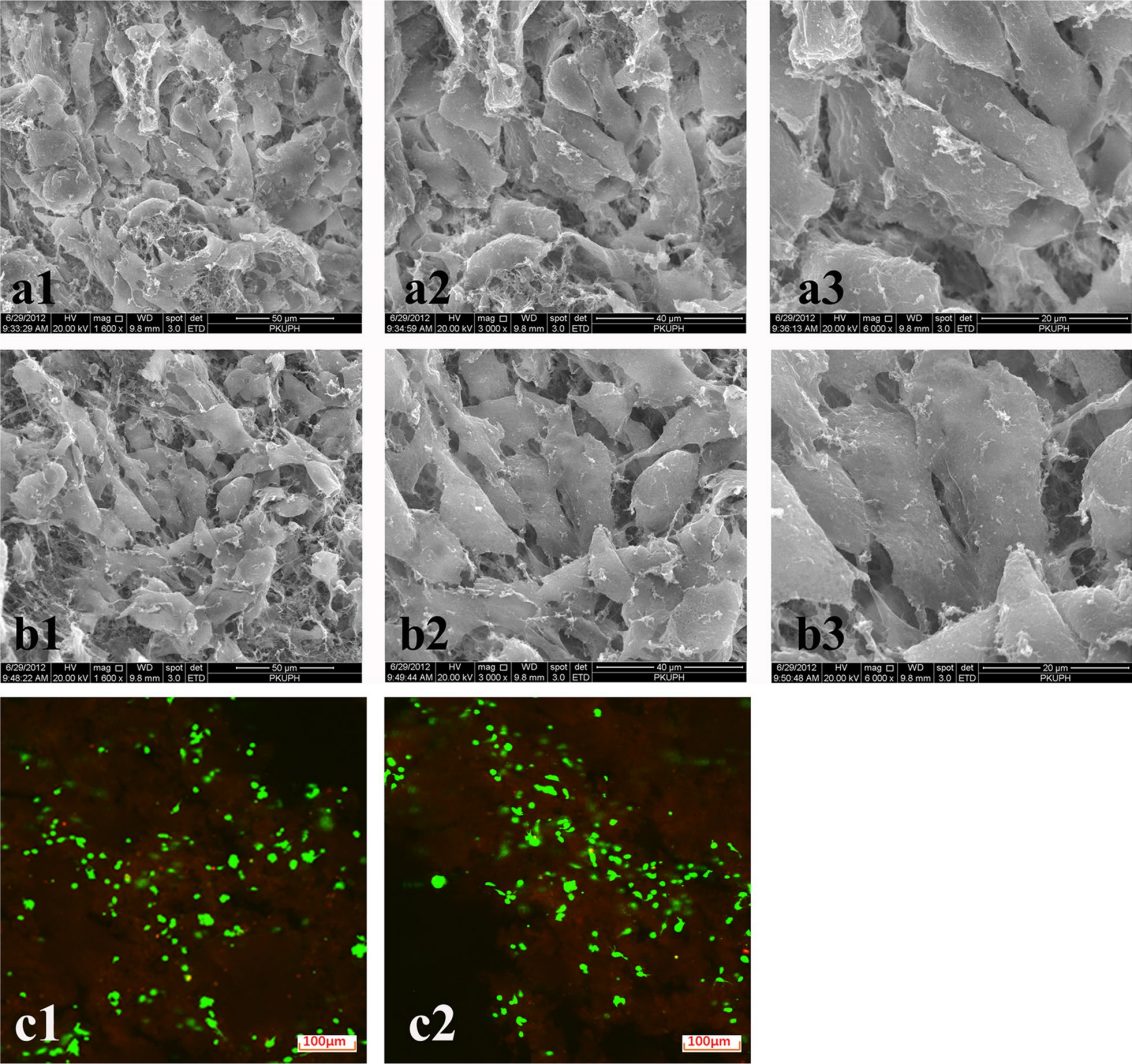
SEM and FCFM images of the ADSCs on the surface of and inside the hydrogel in after 14 days of culture. **a1**–**a3** SEM images of ADSCs on the surface of SNVI-RADA16 hydrogel at a magnification of 1600 × , 3000 × , and 6000 × . **b1**–**b3** SEM images of ADSCs on the surface RADA16 hydrogel at a magnification of 1600 × , 3000 × , and 6000 × . **c1** FCFM image of ADSCs inside SNVI-RADA16 hydrogel (100 ×). **c2** FCFM image of ADSCs inside RADA16 hydrogel (100 ×). Live cells (more than 90%) are stained green, and dead cells (less than 10%) are stained red

We found that ADSCs seeded in the RADA16 and functionalized SNVI-RADA16 hydrogels and cultured in the presence of 10 ng TGF-β have a more ECM retention than monolayer ADSCs cultured with 10 ng TGF-β. This reason may be a high retention effect due to the hydrogel. As indicated by the SEM images in Fig. 4, the ADSCs could tightly adhere to the surface nanofibers in both RADA16 and SNVI-RADA16 hydrogel scaffolds by stretching a large number of pseudopodia. We observed ADSCs with spindle cell as well as polygonal cell morphologies. In addition, a large amount of extracellular matrix was found around the cells. The FCFM images in Fig. 4 show, at 14 days after culture, a large amount of ADSCs (more than 90%) are stained green (live cells), and a few (less than 10%) are stained red (dead cells) in the RADA16 and SNVI-RADA16 hydrogels. This result showed that a large amount of ADSCs was able to survive on the surface and the inside of the hydrogels. However, there was no significant difference between the number of viable cells in the RADA16 and SNVI-RADA16 hydrogels.

### Detection of collagen type I and II, aggrecan and HIF-1 mRNA by real-time PCR

**Fig. 5:**
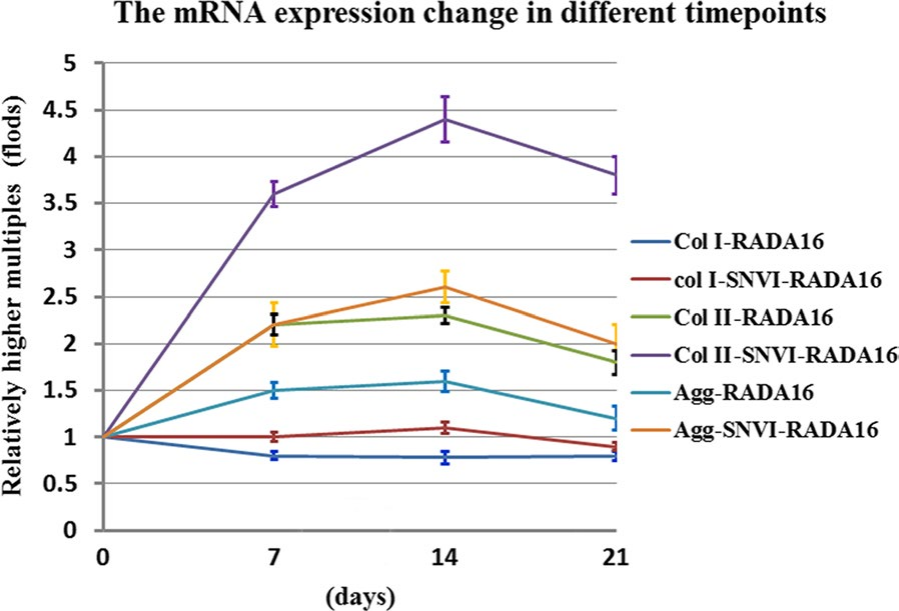
mRNA expression of collagen type I and II, and aggrecan compared to the control group in RADA16 and SNVI-RADA16 hydrogels after 7, 14, and 21 days in culture. Col I, collagen type I; Col II, collagen type II; Agg, aggrecan

To assess the effects of the different peptides on the ADSCs, the mRNA levels of collagen type I and II, and aggrecan were assayed by real-time PCR at 7, 14, and 21 days of culture. As shown in Fig. 5, from 0 to 21 days, the mRNA expression of collagen type II and aggrecan significantly increased in the cells grown in the RADA16 and SNVI-RADA16 hydrogel scaffolds in comparison to the control cells (*p* < 0.001). The increase was higher in the SNVI-RADA16 than in the RADA16 scaffold at all time points. The stimulating effect was mainly observed during the first 14 days, after which the expression slightly decreased again. The expression of collagen type I was not significantly different from that in the control (*p* > 0.05).

### Gene expression HIF-1α

**Fig. 6:**
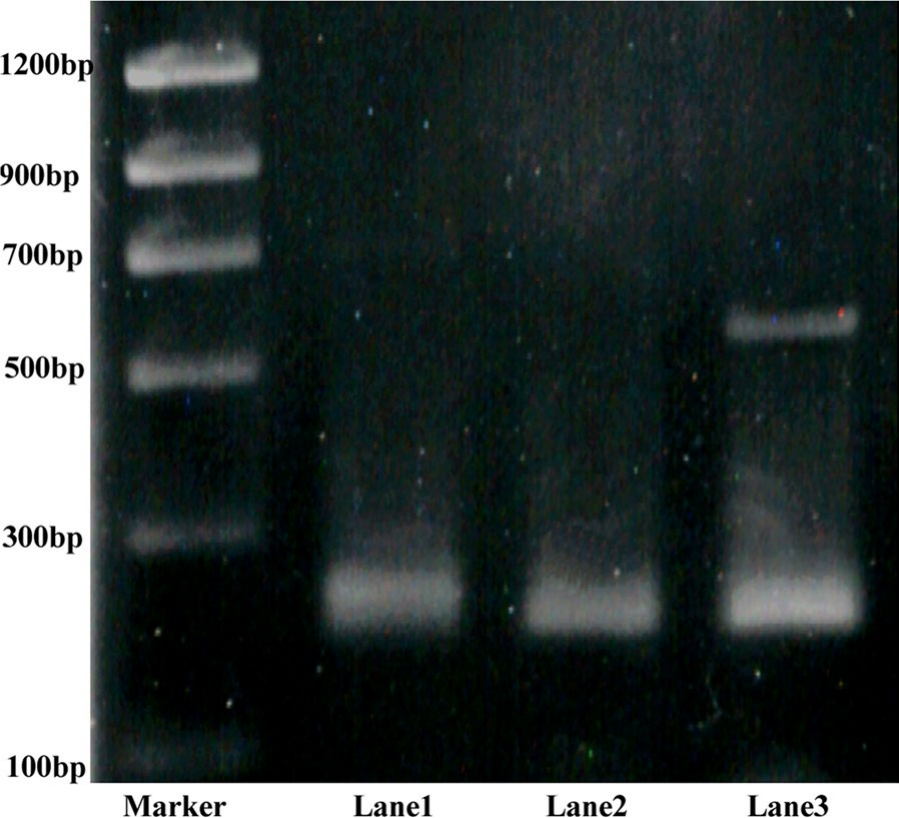
RT-PCR for canine HIF-1α mRNA. Lane 1 for the monoculture ADSCs, Lanes 2 and 3 for RADA 16 and SNVI-RADA16 hydrogels at 21 days culture, respectively. Lane 3 shows the cells in SNVI-RADA16 hydrogels have expressed the target gene HIF-1α mRNA

At 21 days culture, we found that ADSCs in SNVI-RADA16 hydrogels expressed the HIF-1a mRNA by RT-PC. However, HIF-1 mRNA is absence in control gel and monolayer. The expression of the GAPDH gene, which was included as an internal control, can be detected in all groups (Fig. 6).

### ELISA of collagen type I and II, and aggrecan

**Fig. 7:**
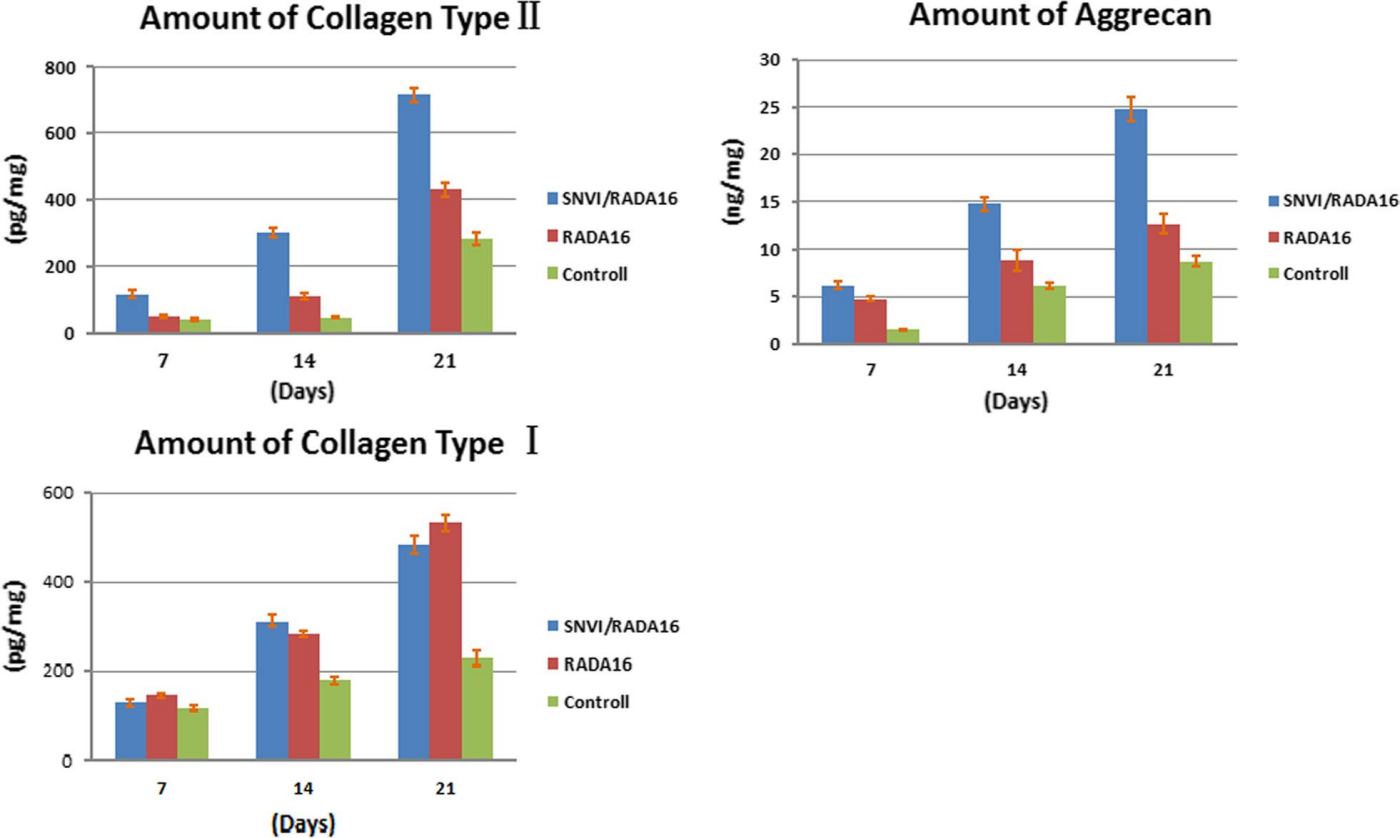
ELISA of collagen type I (**a**) and II (**b**), and aggrecan (**c**) compared to the control group in EMC after 7, 14, and 21 days in culture

To assess the effects of the different peptides on the ADSCs further, collagen type I and II, and aggrecan were assayed by ELISA at 7, 14, and 21 days. As shown in Fig. 7, from 0 to 21 days, collagen type II and aggrecan were obviously increased in RADA16 and SNVI-RADA16 hydrogel scaffolds in comparison to the control (*p* < 0.001). At all three time points, the expression of collagen type II was higher in cells grown in SNVI-RADA16 than in RADA16 scaffolds. The aggrecan amount was higher in cells from the SNVI-RADA16 scaffold than in those from the RADA16 scaffold at 14 and 21 days of culture. The amount of collagen type I was not significantly different between RADA16 and SNVI-RADA16 at all time points (*p* > 0.05), but it was significantly higher in both scaffold types than in the control cells at 14 and 21 days of culture (*p* < 0.05). The ELISA results showed that the ADSCs in the SNVI-RADA16 scaffold secreted more ECM including collagen type II and aggrecan between 14 and 21 days of culture, than the monolayer ADSCs. The ratio of the aggrecan to collagen is approximately 29:1 in cells in SNVI-RADA16 gel after culture for 21 days.

## Discussion

Tissue engineering has been considered one of the promising treatment strategies for DDD [22–24]. Seeding of cells, growth factors, and scaffolds are three main approaches to engineer NP tissue. In this study, we created a novel functionalized peptide nanofiber scaffold and analyzed its structure and its suitability to grow ADSCs as a putative treatment strategy for DDD.

AFM, CD, and SEM are commonly used to assess the microstructure of peptides, nanofibers, and hydrogels. In this study, we found that while the RADA-SNVI did not allow effective hydrogel formation, both RADA16 and SNVI-RADA mixture hydrogels consisted of uniformly interwoven nanofibers of β-sheet peptides, in consistence with previous studies [13]. The RADA16 and functional peptide solutions self-assembled into dense 3D nanofiber networks with approximately 40-nm diameter and 5–200 nm pore sizes, and contained over 99 % water content, which was in accordance with previous studies [25, 26]. 3D structure would have an important effect on cell morphology and function. Although the SNVI-RADA16 and RADA16 hydrogels have a different nanofiber diameter, the SNVI-RADA16 hydrogel has the same porosity as the RADA16 hydrogel. We think that the porosity of scaffold have a more important effect on cell morphology and function.

Gelain et al. [13] reported that the addition of motifs containing up to 12 additional residues to the self-assembling peptide RADA16 did not inhibit the self-assembly and nanofiber formation. In contrast, our results showed that the SNVI peptide of 10 amino acids long did significantly inhibit the self-assembly and nanofiber formation of the RADA16 peptides. This might be explained by the difference in electric charge of various amino acids, which can affect the secondary structure of the protein.

The biocompatibility of the scaffold is of critical importance for tissue engineering. Our results showed that the ADSCs could tightly adhere to the surface of the nanofibers in both RADA16 and SNVI-RADA16 hydrogel scaffolds by stretching a large number of pseudopodia, and effectively colonized the entire scaffolds. These results suggested that RADA16-based hydrogel scaffolds maintain the ADSC viability. In addition, the peptides were synthesized with amino acids that are completely biodegradable in the body [27]. Taken together, these results suggest that the RADA16-based scaffolds confer good biocompatibility for ADSCs.

In addition, we investigated whether the ADSC-seeded hydrogels mimicked the biological function of the NP. Collagen type I and II, and aggrecan are used as NP and cartilage markers [28–31]. We found that the mRNA expression of collagen type II and aggrecan, but not of collagen type I, was significantly increased in RADA16 and SNVI-RADA16 hydrogel scaffolds in comparison to the control group. The increased expression of both proteins between 7 and 14 days of culture indicate a stimulating effect of TGF-β and the SNVI peptide. The decline in expression after 14 days of culture might indicate the presence of a feedback mechanism induced by the overexpression of collagen type II and aggrecan.

In our study, we found that ADSCs in SNVI-RADA16 gels expressed the hypoxia-inducible factor 1a (HIF-1a) mRNA by RT-PCR at 21 days culture. However, HIF-1 mRNA is absence in control hydrogel and monolayer. Furthermore, the ELISA results showed that the ADSCs in the SNVI-RADA16 scaffold secreted more ECM including collagen type II and aggrecan between 14 and 21 days of culture, than the monolayer ADSCs. The ratio of the aggrecan to collagen type II is approximately 29:1 in the NP after culture for 21 days. Our results are consistent with those reported by Mwale et al. [31, 32]. Mwale’s study showed that the ratio of aggrecan to collagen type II in the NP of young adults is approximately 27:1, whereas the ratio within the hyaline cartilage endplate is approximately 2:1. According to the JOR’s reference [32] in 2015, we think that cells in SNVI-RADA16 gel after culture for 21 days are the nucleus pulposus-like cells, are significant difference to chondrocyte. Therefore, our results suggested that the ADSC-seeded SNVI-RADA16 scaffold had NP-like biological properties. Compared to the ADSC-seeded RADA16 matrix, the hBMP-7-functionalized peptides promoted the differentiation of ADSCs into cells with an NP-like phenotype.

Cells could secrete more ECM when grown in a 3D culture environment [33–35]. Our results are consistent with this finding. The expression of the cartilage markers in RADA16 and SNVI-RADA16 hydrogels was significantly higher than in the monolayer cells after 14 and 21 days in culture. We hypothesize that the peptide hydrogels with a 3D microstructural architecture provide more space for the ADSCs to attach and proliferate. In addition, our results showed that the ADSCs secreted more ECM in the SNVI-RADA16 than in the RADA16 hydrogel, suggesting that the short peptide SNVI promotes ECM secretion.

The short synthetic peptide SNVI consists of 10 amino acids and originates from the bioactive area of hBMP-7. A previous study demonstrated that the peptide enhanced osteoblast adhesion, spreading, and differentiation [21]. We found that ADSCs seeded in the RADA16 and functionalized SNVI-RADA16 hydrogels and cultured in the presence of 10 ng TGF-β secreted more ECM than monolayer ADSCs cultured with 10 ng TGF-β. Moreover, we found that cells cultured in the SNVI-RADA16 hydrogel showed higher expression of collagen type II and aggrecan than those grown in the RADA16 hydrogel after 14 and 21 days of culture, which indicates higher ECM secretion. We hypothesize that the increased ECM secretion in the SNVI-RADA16 hydrogel was due to the SNVI peptide with hBMP-7 activity, suggesting that it plays an important role in promoting ECM secretion. Furthermore, we can conclude that the functionalized self-assembling peptide with hBMP-7 biological activity promoted the differentiation of ADSCs into NP-like cells.

## Conclusions

In this study, we conjugated the short peptide motif SNVI to the C-terminus of the self-assembling peptide RADA16 to obtain a novel functionalized self-assembling peptide RADA16-SNVI, which have the BMP7 biological activity. Our study demonstrated that the mixture of the RADA16 and RADA16-SNVI promoted the differentiation of the ADSCs into NP-like cells. Thus, the hydrogel of the mixture peptides may be suitable for application in NP tissue regeneration.

## Abbreviations

DDD: Disc degeneration disease
IVD: Intervertebral discs
NP: Nucleus pulposus
ECM: Extracellular matrix
TGF- β: Transforming growth factor-β
BMP-7: Bone morphogenetic protein-7
hBMP-7: Human bone morphogenetic protein-7
HPLC: High-performance liquid chromatography
AFM: Atomic force microscopy
CD: Circular dichroism
SEM: Scanning electron microscopy
ADSCs: Adipose tissue-derived stem cells
Ct: Threshold cycles
HIF-1α: Hypoxia-inducible factor-1α
ELISA: Enzyme-linked immunosorbent assay
SD: Standard deviation
